# Correction: Prevalence and associated risk factors for mental health problems among patients with polycystic ovary syndrome in Bangladesh: A nationwide cross-sectional study

**DOI:** 10.1371/journal.pone.0304879

**Published:** 2024-05-31

**Authors:** Moynul Hasan, Sumaya Sultana, Md. Sohan, Shahnaj Parvin, Md. Ashrafur Rahman, Md. Jamal Hossain, Mohammad Saydur Rahman, Md. Rabiul Islam

The title of the article was incorrectly formatted. The correct formatted title reads as follows: “Prevalence and associated risk factors for mental health problems among patients with polycystic ovary syndrome in Bangladesh: A nationwide cross-sectional study”.

In the Loneliness scale subsection of Materials and methods, there are errors in the paragraph. The correct paragraph is: To calculate the degree of loneliness among respondents, we used the UCLA-3 item loneliness scale. It contains three questions- "How often do you feel?" at the beginning of each question. Lack of companionship, feeling left out, and being isolated from others were the topics of the questions. Here, the respondents had three options to respond- ‘Hardly ever’, ‘Some of the time’, and ‘Often’ with scores 1, 2, and 3, respectively. Then the scores for each question were summed together to measure the loneliness status of the respondents. The higher scores indicate severe loneliness and vice versa [43].

The reference 43 is incorrect. The correct reference is: Steptoe A, Shankar A, Demakakos P, Wardle J. Social isolation, loneliness, and all-cause mortality in older men and women. Proc Natl Acad Sci U S A. 2013;110(15):5797–5801. doi:10.1073/pnas.1219686110.
