# Supplementary material for: Prevalence and associated risk factors for mental health problems among patients with polycystic ovary syndrome in Bangladesh: A nationwide cross—Sectional study
Source: PLoS One. 2022 Jun 22;17(6):e0270102. doi: 10.1371/journal.pone.0270102 (PMC9216580; doi:10.1371/journal.pone.0270102)
Supplement: Attachment — Submitted filename: review1.docx [file pone.0270102.s001.docx]

This is a valuable study about the mental health problem of PCOS women from the perspective of socio-demographic profile and lifestyle-related factors in Bangladesh.

However, there are some comments to the author.

1. What is the novelty of this study? Is it only that it is the first study in Bangladesh? The author should clearly state the novelty.
2. What is the message in Figure 1? The author should provide a discussion comparing the results with previous reports.
3. In Results, the description “Regression analysisのRespondents who took morning or afternoon snacks sometimes were more likely to have the depressive disorder than the respondents who never took snacks (OR=2.00, 95% CI 0.75-5.32, p=0.007)” is inappropriate because the 95% CI straddles 1.
4. In Discussion, there is the description “According to our analysis, from the perspective of the socio-demographic profile, we concluded that marital status, education, financial background, area of residence, smoking habit, and family history of PCOS might be responsible for developing mental health issues among our participants.” More consideration should be given to each of these factors. How does this compare to previous reports? The background of significant differences in each factor should be examined in detail.
5. In Discussion, repeated mention of the result is not required. “About 59.86%, 62.40%, and 57.79% of participants with low economic backgrounds were suffering from loneliness, anxiety, and depressive disorder. Almost half of the women among our participants have complained about their married life which was responsible for their poor mental health. According to our findings, women living in urban area (loneliness=79.58%, generalized anxiety disorder=78.67%, depressive disorder=78.69%), having no smoking habit (loneliness=95.85%, generalized anxiety disorder=95.54%, depressive disorder=95.08%), and having no family history of PCOS (loneliness=53.74%, generalized anxiety disorder=81.06%, depressive disorder=79.51%) might be more at risk of mental health problems.”
6. In Discussion, what is the meaning of a, b and c in the description “As per our analysis, the a%, b%, and c% of respondents are struggling with loneliness, anxiety, and depression, respectively”?
