# Supplementary material for: Prevalence and associated risk factors for mental health problems among patients with polycystic ovary syndrome in Bangladesh: A nationwide cross—Sectional study
Source: PLoS One. 2022 Jun 22;17(6):e0270102. doi: 10.1371/journal.pone.0270102 (PMC9216580; doi:10.1371/journal.pone.0270102)
Supplement: Attachment — Submitted filename: Final Comments EMID.docx [file pone.0270102.s002.docx]

Dear Editor

I observed that authors complied all the remarks and thus recommending for the publication.

Thank you for giving this opportunity.

Best Regards
